# A Microactuator Array Based on Ionic Electroactive Artificial Muscles for Cell Mechanical Stimulation

**DOI:** 10.3390/biomimetics9050281

**Published:** 2024-05-08

**Authors:** Jing Gu, Zixing Zhou, Yang Xie, Xiaobin Zhu, Guoyou Huang, Zuoqi Zhang

**Affiliations:** 1 Department of Engineering Mechanics, School of Civil Engineering, Wuhan University, Wuhan 430072, China; 2017301890005@whu.edu.cn (J.G.); 2018302100256@whu.edu.cn (Z.Z.); treuseflorian689@gmail.com (Y.X.); 2 Department of Spine Surgery and Musculoskeletal Tumor, Zhongnan Hospital of Wuhan University, Wuhan 430072, China; xiaobinzhu@whu.edu.cn

**Keywords:** microactuator, artificial muscle, electroactive polymer, electro-actuation, biomechanics

## Abstract

Mechanical stimulation is prevalent within organisms, and appropriate regulation of such stimulation can significantly enhance cellular functions. Consequently, the in vitro construction and simulation of mechanical stimulation have emerged as a research hotspot in biomechanics. In recent years, a class of artificial muscles named electroactive polymers (EAPs), especially ionic EAPs, have shown promising applications in biomechanics. While several techniques utilizing ionic EAPs for cell mechanical stimulation have been reported, further research is needed to advance and enhance their practical applications. Here, we prepared a microactuator array based on ionic EAP artificial muscles for cell mechanical stimulation. As a preliminary effort, we created a 5 × 5 microactuator array on a supporting membrane by employing laser cutting. We evaluated the electro-actuation performance of the microactuators through experimental testing and numerical simulations, affirming the potential use of the microactuator array for cell mechanical stimulation. The devised approach could inspire innovative design concepts in the development of miniaturized intelligent electronic devices, not only in biomechanics and biomimetics but also in other related fields.

## 1. Introduction

The organism experiences widespread mechanical stimulation, which plays a critical role in various physiological processes [1]. Research has demonstrated that mechanical stimulation profoundly influences cellular behavior, development, repair, and regeneration, among other factors, thereby impacting the overall growth and development of the organism [2,3,4]. Effective regulation of mechanical stimulation holds great potential for enhancing cellular functionality. Consequently, the construction and simulation of mechanical stimulation in vitro have emerged as prominent research areas within the field of biomechanics, leading to the development of numerous materials, devices, and technologies aimed at appropriately providing mechanical stimulation to cells [5,6,7,8].

In recent years, there has been significant research interest in the field of biomechanics toward electroactive polymers (EAPs). EAPs are a novel class of flexible smart materials that typically exhibit shape or size changes under electrical excitation. They are also known as artificial muscles due to the similarity between the actuation deformation characteristics of EAPs and biological muscles. EAPs can be broadly categorized into electronic EAPs and ionic EAPs. Electronic EAPs primarily include dielectric elastomers, ferroelectric polymers, electrostrictive paper, electrostrictive graft elastomers, liquid crystalline elastomers, etc., while ionic EAPs mainly include conducting polymers, ionic polymer–metal composite, carbon nanotubes, ionic polymer gels, etc. EAPs possess advantages such as large deformation, light weight, and ease of processing. Compared to electronic EAPs, ionic EAPs have the advantage of lower actuation voltages (typically less than 10 V). Soft actuators based on ionic EAPs have found extensive applications in biomedicine [9,10,11], biomimetic machinery [12,13,14], micro-electro-mechanical systems [15,16,17], and more. Meanwhile, they are gradually demonstrating significant potential applications in biomechanics. Recently reported cell mechanical stimulation technologies based on ionic EAPs have showcased the utility of such materials, along with their compatibility with cell culture [18,19,20]. Nevertheless, further research is needed to advance and enhance their practical applications.

**Figure 1:**
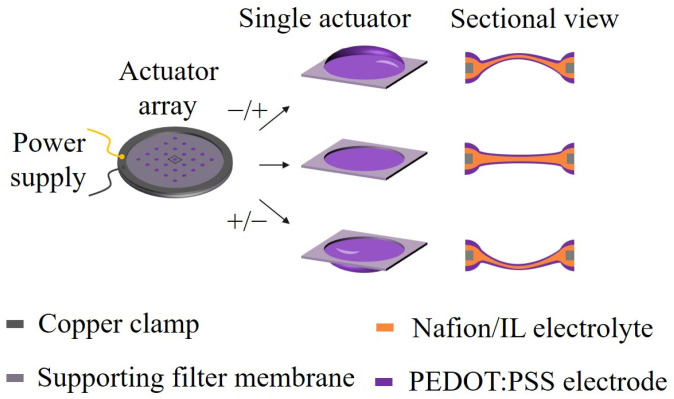
Schematic illustrations of the microactuator array based on ionic EAP artificial muscles for cell mechanical stimulation. A 5 × 5 circular hole array with a diameter of 1.5 mm was designed on the substrate membrane using the laser cutting method. Nafion/ionic liquid and PEDOT:PSS were chosen as the core layer and electrode materials of the microactuators, respectively. Under a small electrical excitation, the microactuators can produce a controllable out-of-plane deformation response.

In this work, we prepared a microactuator array based on ionic EAP artificial muscles for cell mechanical stimulation (Figure 1). As a preliminary attempt, we designed a 5 × 5 circular hole array with a diameter of 1.5 mm on a supporting substrate membrane using laser cutting. Subsequently, we selected Nafion/ionic liquid and poly(3,4-ethylenedioxythiophene)/poly(styrenesulfonate) (PEDOT:PSS) as the core layer and electrode materials of the microactuators, respectively, to create a 5 × 5 microactuator array. Under a small electrical excitation, the actuators can produce a controllable out-of-plane deformation response. We studied the performance of the microactuators through experimental tests and numerical simulations, and explored the potential of using the microactuator array for cell mechanical stimulation.

## 2. Materials and Methods

### 2.1. Preparation of the Microactuator Array

**Figure 2:**
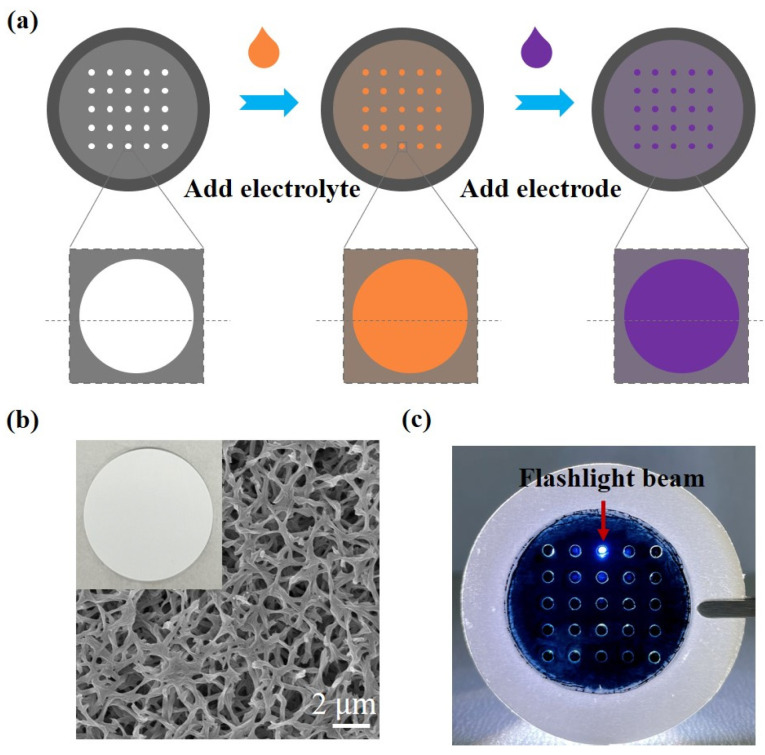
Fabrication and structural characterization of the microactuator array. (**a**) Fabrication process of the microactuator array; (**b**) representative SEM micrograph of the surface morphology of the substrate membrane; (**c**) image of the microactuator array under flashlight illumination.

In this study, the performance requirements of the supporting substrate material for fabricating the microactuator array mainly include good resistance to organic solvents and sufficient mechanical strength. The filter membranes are readily available to us and are relatively low in cost, making them the primary consideration when selecting the substrate material. After testing and comparing several common filter membranes, we found that the preparation of the microactuator array on the nylon filter membrane was relatively successful, and both the cured Nafion and PEDOT:PSS could securely adhere to the substrate membrane. Therefore, we believe that this filter membrane can meet our research needs adequately. A nylon filter membrane (Chuangwei filtration) with a diameter of 47 mm and a pore size of 0.1 µm was chosen as the supporting substrate for fabricating the microactuator array (Figure 2a). A 5 × 5 circular hole array with an aperture of 1.5 mm was cut out on the membrane using a laser cutter (Ketai 3020) and cleaned with ultra-pure water. A microactuator will be generated at each circular hole. Nafion/ionic liquid is the core material for the microactuators, and the solution was formulated as follows: a certain volume of Nafion solution (Chemours D2020, 20%, Wilmington, DE, USA) was added into the glass bottle and dried at 70 °C for one day; N,N-Dimethylacetamide (DMAC; Sigma-Aldrich, St. Louis, MO, USA), twice the volume of Nafion solution, was then added to dissolve Nafion. The mixture was stirred in a water bath at 60 °C for one day with a rotating speed of 300 r/min. Subsequently, 1-ethyl-3-methylimidazolium tetrafluoroborate (EMImBF4; Sigma-Aldrich, ≥98% (HPLC)) ionic liquid (IL) was added in a mass ratio of 1:0.6 to Nafion. The mixture was stirred in a water bath at 45 °C for one day at a rotating speed of 300 r/min. The substrate membrane containing the circular hole array was fully soaked in the Nafion solution to ensure that all holes were filled with Nafion. Afterward, the membrane was clamped by two Teflon ring plates and dried at 40 °C for one day in an upright position for the initial curing of Nafion. Then, the membrane was dried at 80 °C for 4 h to further solidify Nafion and enhance its mechanical strength.

PEDOT:PSS was chosen as the electrode material for the microactuators. To enhance its conductivity, 5% dimethyl sulfoxide (DMSO) was added to the PEDOT:PSS solution (Heraeus, CLEVIOS PH 1000, Hong Kong, China). Subsequently, pure water, four times the volume of PEDOT:PSS, was added to dilute the solution. A volume of 1 mL of the PEDOT:PSS solution was added to the surface of the substrate membrane containing Nafion film and dried at 60 °C for 2 h, resulting in the deposition of a layer of PEDOT:PSS. The sample was then flipped, and the above operation was repeated on the other side. Ultimately, a 5 × 5 microactuator array, consisting of Nafion/IL and PEDOT:PSS, was obtained.

### 2.2. Structural and Electrical Characterization

A scanning electron microscope (SEM; Zeiss SIGMA, Oberkochen, Germany) was employed to observe the surface morphology of the substrate membrane and the cross-sectional structures of the microactuators at a scanning voltage of 2 kV. The surface resistance of the PEDOT:PSS electrodes was measured using a square resistance meter (Four Probe Technology HP-504) to characterize conductivity.

### 2.3. Electro-Actuation Performance Testing

**Figure 3:**
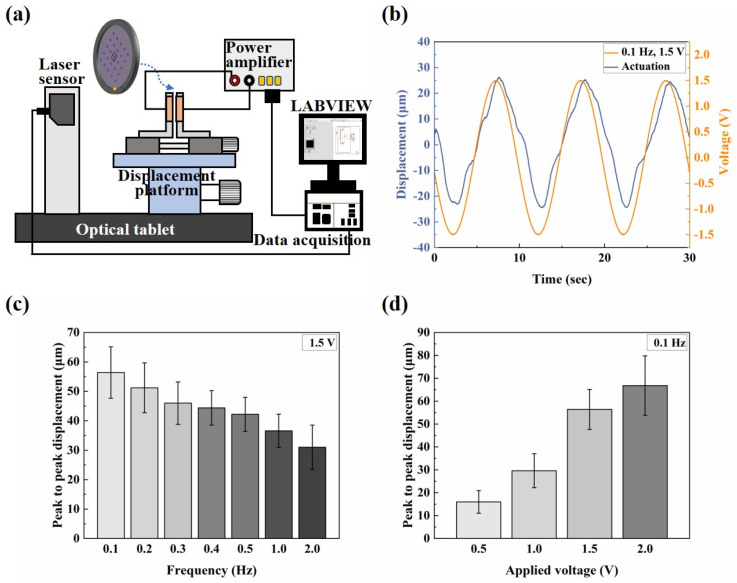
Electro-actuation performance testing of the microactuator array. (**a**) Illustration of the main components of the electro-actuation testing system; (**b**) representative curve of the out-of-plane deformation response of the center point of a microactuator under a sinusoidal voltage of 0.1 Hz 1.5 V; (**c**,**d**) the electro-actuation performance of the microactuators under sinusoidal voltages of (**c**) different frequencies and (**d**) different amplitudes, respectively. The data are shown as mean ± SD.

The electro-actuation performance was characterized by measuring the displacement response of the microactuators under applied voltages. A test system (Figure 3a) was established, comprising a computer equipped with Labview programming, a data acquisition card (NI PCIe-6351), a power amplifier (Aigtek ATA-304), a three-way displacement gripper platform (with a pair of clamps affixed with copper leaf serving as electrodes), and a laser displacement sensor (Keyence IL-065). Labview programming and the data acquisition card were utilized for analog signal generation, simultaneously collecting voltage and displacement data. Since the analog signal itself does not possess the actual actuation function, the primary role of the power amplifier was to convert the analog electrical signal into the actual actuation voltage. The laser displacement sensor was employed for displacement sensing of the microactuators. During the test, two copper rings were used to clamp the sample, positioned between the electrodes. By adjusting the displacement platform, the laser beam of the displacement sensor was directed vertically toward the central point of each microactuator to test the electro-displacement response. Additionally, the performance of ionic polymer microactuators is strongly affected by changes in humidity. Therefore, we installed enclosed chambers around the electro-actuation test platform and conducted our testing within the same ambient humidity range (40~50%RH) whenever possible.

### 2.4. Mechanical Characterization

**Figure 4:**
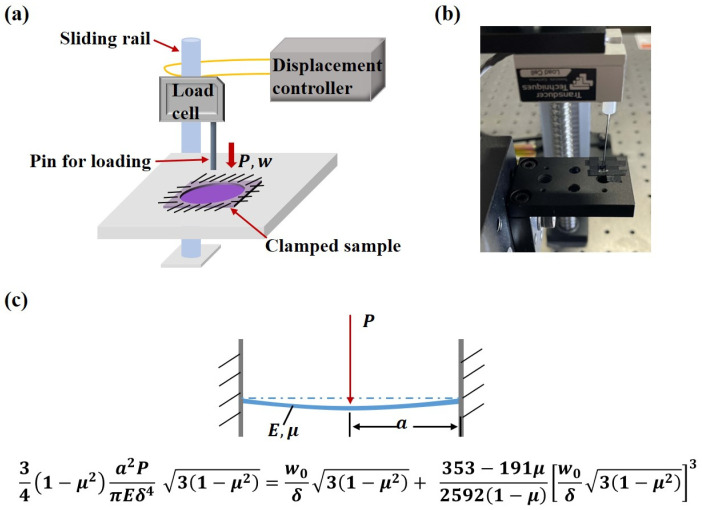
Mechanical characterization of the microactuator array. (**a**) Illustration of the mechanical testing platform; (**b**) image of the mechanical testing platform; (**c**) illustration of the large deflection theory of circular thin plates when the edge is clamped and the center is subjected to a concentrated load P.

The average thickness of the microactuator (~13 µm) is much smaller than its diameter (*d* = 1.5 mm), allowing each microactuator to be treated as an elastic circular thin plate that bends when powered on. The microactuators were closely connected to the substrate membrane around them, making it challenging to separate them for independent tests. Considering the overall structure, deformation characteristics of the microactuator array, and operation simplicity, we designed a platform to subject a single microactuator to out-of-plane deformation and characterize its equivalent elastic modulus (Figure 4a–c). To achieve this, a single microactuator was cut out along with a small portion of the substrate membrane around it, and the microactuator’s edge was securely fastened with highly adhesive tape to a test plate with a round through hole of the same size. A pin, with a diameter much smaller than that of the microactuator, was affixed to the load rod of a load cell (Transducer Techniques GSO-10, Temecula, CA, USA), ensuring precise alignment directly above the center of the microactuator. The load cell, mounted on a sliding rail connected to a displacement controller (Tankon Technology TMC300, Shenzhen, China), allowed the force sensor to move up and down. Throughout the test, the boundary condition of the microactuator was treated as edge clamping, and the force sensor, equipped with the pin, descended slowly at a constant speed of 10 µm/s. Under the transverse load applied by the pin, the microactuator bent, and the displacement of the pin was equal to that of the center point of the microactuator. Thus, the load–displacement relationship of the center point of the microactuator was obtained. Subsequently, the equivalent elastic modulus of the microactuator was calculated using circular thin plate bending theory.

According to the large deflection theory of circular thin plates, when the edge of a circular thin plate is clamped and the center is subjected to a concentrated load *P*, by the perturbation method [21,22], we have

(1) $$\frac{3}{4}\left(1-\mu^{2}\right)\frac{a^{2}P}{\pi E\delta^{4}}\sqrt{3\left(1-\mu^{2}\right)}=\frac{w_{0}}{\delta }\sqrt{3\left(1-\mu^{2}\right)}+\frac{353-191\mu }{2592\left(1-\mu \right)}{\left[\frac{w_{0}}{\delta }\sqrt{3\left(1-\mu^{2}\right)}\right]}^{3},$$

where μ is the Poisson’s ratio, a is the radius of the plate, E is the equivalent elastic modulus, δ is the thickness of the plate, and $w_{0}$ is the deflection of the center of the plate.

### 2.5. Numerical Simulation

When the microactuator is energized, the gain and loss of electrons are accompanied by the redox reaction of PEDOT in the electrodes. To maintain electrical neutrality, ions in the core layer migrate between the electrodes and the electrolyte, causing local volumetric expansion and contraction of the material. Gradient stress and strain occur within the material, inducing the actuator to bend at the macroscale level [23,24,25]. Based on this understanding of the deformation mechanism of the microactuators, we utilized COMSOL Multiphysics 6.0 software to conduct finite element simulation of the electro-deformation response of the microactuators, aiming to validate the experimental phenomenon and provide insights for predicting the electro-actuation performance of materials in future structural design. Three physical modules—transport of dilute species, electric currents, and solid mechanics—were utilized in the model [26]. The first two modules were combined to obtain ion concentrations in different parts of the material at different times of electrification. Subsequently, a coupling coefficient α was applied to the change in ion concentration to represent the stress applied, causing local expansion and contraction of the material in response to the electrochemical behavior of PEDOT. The ion concentration solution was coupled to the solid mechanics module, and the deformation response of the microactuator was determined using linear elastic theory. The main governing equations for each module are as follows:

(2) $$\text{Transport of dilute species:}\;\frac{\partial c_{i}}{\partial t}+\nabla \cdot N_{i}=R_{i},\;N_{i}=-D_{i}\nabla c_{i}-\frac{z_{i}F}{RT}D_{i}c_{i}\nabla V$$

(3) $$\text{Electric currents:}\;\nabla \cdot J=Q_{j,v},\;J=\kappa E+\frac{\partial D}{\partial t}+J_{e},\;E=-\nabla V$$

(4) $$\text{Solid mechanics:}\;\sigma_{ij,j}+f_{i}-\rho {\ddot{u}}_{i}=0$$

where $c_{i}$ represents ion concentration, $N_{i}$ is the ion flux, $R_{i}$ is the reaction rate, $D_{i}$ is the diffusion coefficient, $z_{i}$ is the number of charges per ion, F is the Faraday constant, R is the gas constant, T is the temperature, V is the electric potential, J is the current density, $Q_{j,v}$ is the charge density, κ is the electrode conductivity, D is the electric displacement, E is the electric field, $J_{e}$ is the external current density, $\sigma_{ij}$ represents stress within the material, $f_{i}$ denotes body force, ρ is the material density, and $u_{i}$ stands for displacement.

The diffusion coefficient of the cation was set as $4.2\times 10^{-11}m^{2}/s$ [27] and the material density and relative dielectric constant were set as 1179.8 kg/m^3^ and 1.244 × 10^11^, respectively [13]. Other key parameters will be described in Section 3.

### 2.6. Cell Culture

To establish a biocompatible environment for subsequent cell culture while isolating the influence of electric stimulation on cells, we coated the microactuators with polydimethylsiloxane (PDMS; Dow Corning Toray, Midland, TX, USA). The PDMS precursor, with a mass ratio of 10:1 for the monomer and crosslinker, was prepared and degassed. It was then evenly spread on the microactuators using a spinning coater (LEBO Scientific Instrument EZ4-S-PP, Wuxi, China) at a rotational speed of 1500 r/min for 500 s. Subsequently, the sample was dried at 40 °C to solidify the PDMS. Later, we utilized the test platform described in Section 2.3 to examine the electro-actuation performance of the PDMS-coated microactuator array, aiming to verify the feasibility of this structure as a cell mechanical stimulation device. The PDMS-coated microactuators were tested within the supporting substrate, using the same approach as the microactuators without PDMS.

For cell culture, the microactuator array was cleaned with ultra-pure water and immersed in a collagen solution prepared with PBS buffer (Solarbio Tablets, Beijing, China) at a concentration of 15 µg/mL for one day. Subsequently, the microactuator array was rinsed with phosphate buffer saline (PBS) buffer and sterilized with UV light in a super clean bench for 2 h. Following sterilization, a NIH/3T3 cell suspension with a cell concentration of 5 × $10^{4}$ cells/mL was applied onto the microactuator surface. The cells were cultured in Dulbecco’s modified Eagle medium (DMEM; Gibco/Thermo Fisher Scientific, Waltham, MA, USA) with 10% fetal bovine serum (FBS; Newzerum, Christchurch, New Zealand) and 1% pen/strep (Gibco/Thermo Fisher Scientific). Culturing took place in an incubator (Heal Force HF90) at 37 °C with 5% CO_2_. The morphological changes of the cells were observed and documented daily using a microscope (Olympus CKX5).

## 3. Results and Discussion

We compared several common substrate membranes and chose a nylon filter membrane with a diameter of 47 mm and a pore size of 0.1 µm as the supporting substrate for creating a microactuator array. Figure 2b clearly shows the porous structure of the filter membrane. Next, we employed a laser cutter to create a 5 × 5 circular hole array with an aperture of 1.5 mm on the membrane [28]. Since the circular hole array can basically meet the requirements of subsequent experiments, we believe that the influence of laser cutting on the material in this experiment is within the tolerable range. The cutting pattern was controlled by computer drawing. Therefore, a major advantage of this method is that almost any shape, number, and formation of microactuator patterns can be designed on the substrate material according to the needs of actual research and application.

Nafion/IL serves as the core material for the microactuators. The Nafion film exhibits characteristics such as high proton conductivity, good chemical stability, and excellent mechanical properties [29]. After the Nafion curing process, the film demonstrated good elasticity when lightly touched. PEDOT:PSS, chosen for its excellent electrical conductivity and flexibility, functions as the electrode material for the microactuators. Following the preparation, it was observed that the microactuators were securely connected to the surrounding substrate membrane, and the deposition of PEDOT:PSS was relatively uniform. Furthermore, the microactuators exhibited some degree of light transmittance under flashlight illumination (Figure 2c).

**Figure 5:**
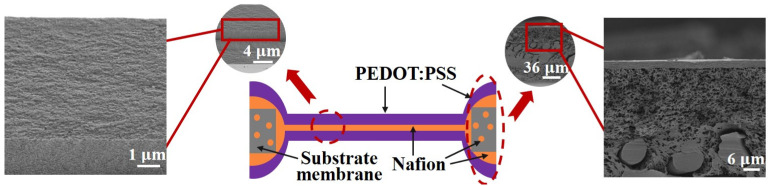
Illustration and SEM micrographs of the cross-sectional structures of the microactuators.

Figure 5 presents the schematic diagram and SEM micrographs of the electrode–core interface of the microactuators, along with the cross-section of the substrate material outside the holes. It is evident that the PEDOT:PSS electrode layer closely adhered to both the Nafion core layer and the substrate material outside the holes. Simultaneously, Nafion infiltrated into the pores of the substrate membrane, with no observable PEDOT:PSS infiltration. These results ensure the normal and stable operation of the microactuator array under applied voltages, preventing any risk of short circuit. Also, SEM analysis revealed that the microactuator exhibited an average thickness of approximately 13 µm, with the PEDOT:PSS electrode and the Nafion core having average thicknesses of about 5 µm and 3 µm, respectively. The measured surface resistance of PEDOT:PSS outside the holes was about 6 Ω/sq, and that of the microactuators on the holes was approximately 4 Ω/sq after conversion. These findings provide crucial assurance for the electro-actuation performance of the microactuators.

**Figure 6:**
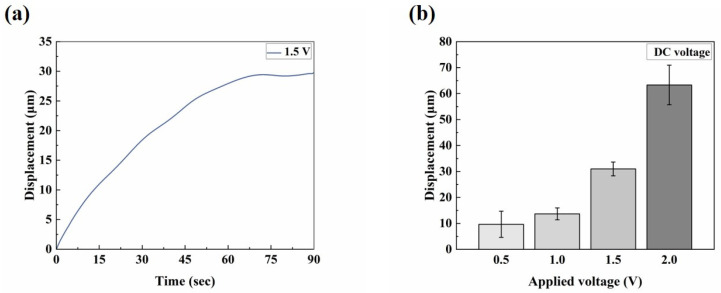
Electro-actuation performance testing of the microactuator array. (**a**) Representative curve of the out-of-plane deformation response of the center point of a microactuator under 1.5 V DC voltage; (**b**) the electro-actuation performance of the microactuators under different DC voltages. The data are shown as mean ± SD.

The electro-actuation performance of the microactuators was primarily characterized by the displacement response of the microactuators under applied voltages. Figure 3a illustrates the main components of the test system. Two copper rings were used to clamp the sample during the test, serving two main purposes: firstly, to impose fixed constraints around the substrate membrane and thereby reduce the interference of other factors, such as the out-of-plane displacement of the membrane itself, on the test results; secondly, the addition of highly conductive copper rings aimed to make the voltage received by the microactuator at each hole on the membrane more uniform, compared to a single-point contact with the electrodes. We applied a sinusoidal voltage to the microactuator array. Figure 3b shows the representative out-of-plane deformation response of the center point of a microactuator under a sinusoidal voltage of 0.1 Hz 1.5 V. The actuator deformed regularly with the change in the external electric field, and the response speed was high. The displacement variation in one cycle was approximately 56 µm. Additionally, we conducted tests on the substrate membrane region outside the holes after electrification. The measured displacement range was small, and there was no obvious regularity (see Figure S1). This fact indicates that the test results of the microactuators can reflect the authentic electro-actuation performance of the microactuator itself. Figure 3c,d show the deformation response of the microactuators under sinusoidal voltages of different frequencies and amplitudes, respectively. The results suggest that the microactuators can produce a significant electro-induced deformation response under a relatively miniaturized size. The deformation degree is related to both the amplitude and frequency of the actuation voltage. Generally, it decreases with the increase in voltage frequency and increases with the increase in voltage amplitude. We also tested the microactuators under different DC voltages, which also demonstrated the good electro-actuation performance of the microactuators (Figure 6a,b).

Firstly, due to the instrument error, we observed that, when actuating the microactuators, the actuation voltage output by the test system was typically very low within approximately 10 s after the Labview signal output program began to run, at around 0.3~0.4 V. It took approximately 10 s to stabilize and align with the set output voltage. This could explain why, when the actuation voltage was set to 1.5 V DC, the microactuators displaced about 2.44 μm at 2.5 s (Figure 6a). As a result of this systematic error, when testing the response of the microactuators under AC excitation, the data collection occurred at least 10 s after initiating the Labview signal output program, representing the stable output stage of the set voltage. Therefore, the results depicted in this figure are also deemed reasonable (Figure 3b). Secondly, the sensitivity of the electro-actuated response of our microactuators under DC excitation may not be as robust as that under sinusoidal voltage excitation, indicating a need for further improvement in our future research endeavors.

**Figure 7:**
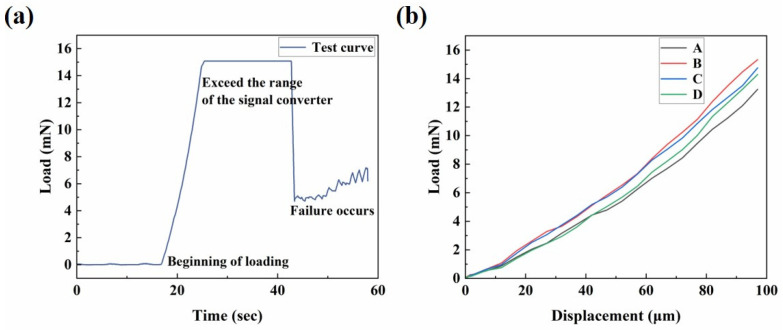
Mechanical characterization of the microactuator array. (**a**) Representative curve of load–time behavior of the center point of a microactuator; (**b**) the load–displacement relationship of several test points in a period of time before failure.

The equivalent elastic modulus is an important parameter used to characterize the performance of the microactuators. Figure 7a presents a representative curve of load–time behavior. The force sensor, equipped with the pin, descended slowly at a constant speed of 10 μm/s. In the initial horizontal stage of the curve, the pin had not yet contacted the microactuator. After contact was established, the pin continued to move downward at a constant speed, and the load gradually increased, eventually leading to the failure of the material. We tested several microactuators to analyze and summarize the general trend of their load–displacement curves. Figure 7b shows the load–displacement relationship of several test points over a period of time before failure. The four curves, labeled A, B, C, and D, represent the test results of different microactuator samples, with identical test methods and locations. During the initial loading stage, when the load was relatively small, the force on the material changed approximately linearly with the displacement. To calculate the equivalent elastic modulus under the large deflection bending theory of circular thin plates (Figure 4c), a pair of load–deflection data was needed. When the deformation was small, the displacement of the center of the actuator was approximately equal to the deflection of the center of the actuator. Therefore, when $w_{0}$ = 2 µm, the mean value of *P* was 0.1858 mN. The estimated Poisson’s ratio of the actuator was 0.38, based on the Poisson’s ratio of Nafion [30,31] and PEDOT:PSS [32]. Finally, the equivalent elastic modulus of the microactuator calculated by the theory was approximately 4.8 GPa. Furthermore, we characterized the Young’s moduli of the constituent materials of the microactuators using atomic force microscopy (AFM; Bruker Dimension Icon, Billerica, MA, USA). The Young’s moduli of PEDOT:PSS and Nafion, as measured by AFM, were approximately 744 MPa and 107 MPa, respectively (refer to Figure S2), demonstrating close agreement with values reported in the literature [33,34].

Based on the electro-actuation testing results, we generally believe that the primary function of the substrate membrane is to support the microactuator array, and its contribution to the electro-actuated deformation of the microactuators is negligible. Therefore, when characterizing the elastic modulus of the microactuators, we primarily consider the mechanical properties of the material itself (PEDOT:PSS-Nafion-PEDOT:PSS), without factoring in the mechanical properties of the substrate membrane.

**Figure 8:**
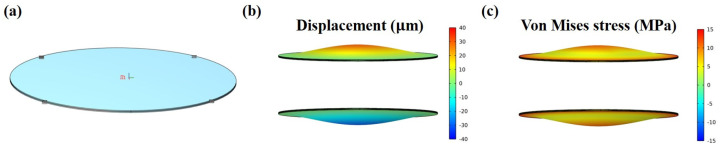
Numerical simulation of the electro-deformation response of the microactuators. (**a**) Demonstration of the object of simulation analysis; (**b**,**c**) illustration of the (**b**) out-of-plane displacement field and (**c**) Von Mises stress field of the microactuator in typical bending states, respectively.

To validate the observed experimental phenomenon and facilitate predictions of the electro-actuation performance in future structural designs, we conducted a finite element simulation of the electro-deformation response of the microactuators. Theoretically, within this microactuator array structure, the material properties and electro-actuation performance of each microactuator are assumed to be identical or very similar. Moreover, they operate independently of each other under applied voltages, akin to parallel circuits. Therefore, in order to improve efficiency, a single microactuator (*d* = 1.5 mm, *h* = 13 μm) was chosen as the object of simulation analysis (Figure 8a). The electric excitation parameters set in the model were the same as those used in the experiment. The out-of-plane displacement field and Von Mises stress field of the material in typical bending states are illustrated in Figure 8b,c, respectively. Within the boundary on the microactuator surface, both displacement and stress exhibited a radial gradient distribution, with the maximum values observed at the center of the material.

**Figure 9:**
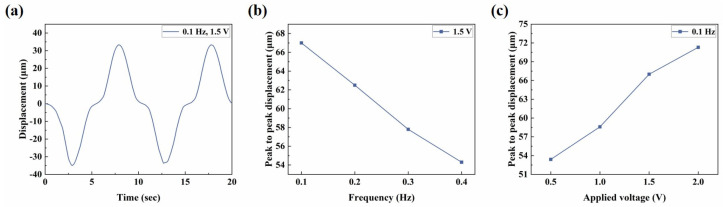
Numerical simulation of the electro-deformation response of the microactuators. (**a**) Simulation curve of out-of-plane deformation of the central point under the excitation of 0.1 Hz, 1.5 V sinusoidal voltage; (**b**,**c**) simulation results of the microactuator deformation response under sinusoidal voltages with (**b**) different frequencies and (**c**) different amplitudes, respectively.

Figure 9a shows the simulation results of out-of-plane deformation of the central point under the excitation of 0.1 Hz 1.5 V sinusoidal voltage. It can be observed that the simulated response curve shared similar regular fluctuation with the test curve, and the displacement variation in one cycle was about 67 µm, slightly larger than the experimental value. Figure 9b,c are the simulation results of the deformation response of the microactuator under sinusoidal voltages with different frequencies and amplitudes, respectively. It can be found that they are similar to the corresponding test results overall, and the trends obtained in the experiment were basically verified. The difference between the simulation and experiment results can be explained from two possible aspects: firstly, due to the structural characteristics of the material, several parameters such as Poisson’s ratio were not directly measured for the time being, and the estimated value may differ from the actual one; secondly, when the bending deformation of the material becomes large, the stiffness of the material may change from that at the initial stage of deformation (Figure 7b), while this model assumed the microactuator as a linear elastic material without considering the influence of nonlinearity.

**Figure 10:**
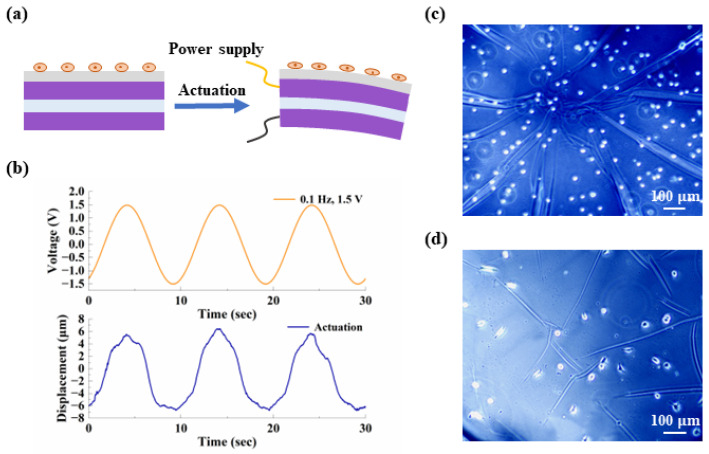
Preliminary validation of the potential for applying the device for cell mechanical stimulation. (**a**) Schematic diagram of a PDMS-coated microactuator. The gray layer in contact with the cells is PDMS; (**b**) representative curves of the out-of-plane deformation response of the center point of a PDMS-coated microactuator under a sinusoidal voltage of 0.1 Hz, 1.5 V; (**c**,**d**) demonstration of the cell morphology (**c**) at the initial time and (**d**) after two days of culture, respectively.

Finally, we added a biocompatible PDMS layer to the existing microactuator array, transforming the structure into a device suitable for cell mechanical stimulation. The working diagram of a single microactuator is shown in Figure 10a. Ideally, when a periodic voltage is applied to the actuator, the reciprocating bending deformation of the microactuator causes the cells above to undergo cyclic stretching. The thickness of the PDMS was measured to be about 40 µm. We applied 0.1 Hz, 1.5 V sinusoidal voltage to the PDMS-modified microactuator array membrane to test the electro-actuation performance. Figure 10b shows the deformation response curve of a test point. It can be observed that the device still produced regular actuation at a fast speed with the change in electric field. The average displacement variation obtained by testing multiple samples was about 14 µm, which was significant, but the deformation was reduced compared with the material without PDMS. According to our analysis, this was likely caused by the large thickness of PDMS. From a mechanical perspective, the addition of PDMS is akin to incorporating a passive deformation layer with considerable mass into the microactuator, thereby reasonably hindering the out-of-plane deformation of the microactuator to some extent. Due to the convenience of operation and the uniformity of coating, we applied a PDMS layer to the microactuator array using the method of spin coating and drying. However, during the experiment, we encountered shortcomings associated with this treatment method. For instance, controlling the amount of PDMS was not straightforward, resulting in the actual thickness of the PDMS layer exceeding our design requirements. Consequently, we plan to refine this experimental step in future studies. Overall, these results indicate that, on a performance level, this microactuator array is expected to meet the requirements of cell mechanical stimulation through proper regulation of relevant parameters.

After the performance test, we cultured NIH/3T3 cells on the microactuators. During cell culture, the microactuators remained in a static state, and the purpose of this step was to verify the device’s biocompatibility and compatibility with cell culture. Figure 10c,d display the cell morphology at the initial time and after two days of culture, respectively. It can be observed that, after two days, the cells exhibited a certain degree of adhesion and spreading compared to the initial state, preliminarily demonstrating the potential for applying the device for cell mechanical stimulation. The next step in our work will focus on achieving a successful combination of cell culture and the electrical signal generation device to ensure the proper functioning of the microactuators.

## 4. Conclusions

In this study, we fabricated a microactuator array based on ionic EAP artificial muscles with the aim of cell mechanical stimulation. Based on the investigations presented in this paper, the following conclusions can be drawn:

(1) Laser cutting technology was employed to conveniently control the shape and formation of the microactuators on the substrate membrane. SEM analysis indicates that the PEDOT:PSS electrode layer closely adhered to both the Nafion core layer and the substrate material outside the holes. Meanwhile, PEDOT:PSS and Nafion exhibited relatively continuous and dense structures. These results validate the reliability of the preparation method used in this study.

(2) The electro-actuation performance of the microactuators was verified through experimental and numerical methods. The experimental results suggest that the microactuators can produce a significant electro-induced deformation response under a relatively miniaturized size. The simulation results are consistent with the corresponding test results overall, and the trends obtained in the experiment were essentially confirmed.

(3) Both the investigation of the electro-deformation response of the PDMS-modified structure and the assessment of the cell behavior cultured on it preliminarily confirmed the feasibility of this structure for cell mechanical stimulation.

We anticipate that the introduction of this microactuator array can inspire novel design concepts for miniaturized intelligent electronic devices, not only in biomechanics and biomimetics but also in other related fields.

## Data Availability

Data are available upon request to the corresponding authors.

## Supplementary Materials

The following supporting information can be downloaded at: https://www.mdpi.com/article/10.3390/biomimetics9050281/s1, Figure S1: Representative curves of the deformation behavior of the substrate membrane region under a sinusoidal voltage of 0.1 Hz, 1.5 V; Figure S2: The Young’s modulus of PEDOT:PSS and Nafion measured by AFM. The data are shown as mean ± SD.
