# Supplementary material for: A Microactuator Array Based on Ionic Electroactive Artificial Muscles for Cell Mechanical Stimulation
Source: Biomimetics (Basel). 2024 May 8;9(5):281. doi: 10.3390/biomimetics9050281 (PMC11117532; doi:10.3390/biomimetics9050281)
Supplement: Supplementary file 1 [file biomimetics-09-00281-s001.zip › biomimetics-2932321-supplementary.pdf]

biomimetics

# A Microactuator Array Based on Ionic Electroactive Artificial Muscles for Cell Mechanical Stimulation

Jing Gu [1], Zixing Zhou [1], Yang Xie [1], Xiaobin Zhu [2], Guoyou Huang [1,*] and Zuoqi Zhang [1,*]

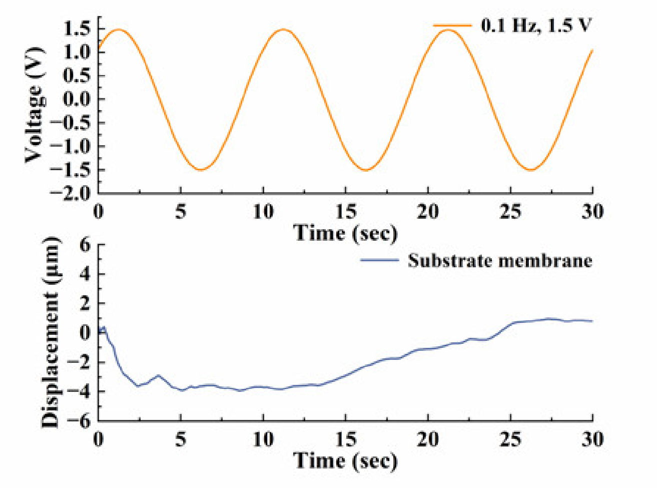


**Figure S1.** Representative curves of the deformation behavior of the substrate membrane region under a sinusoidal voltage of 0.1 Hz 1.5 V.

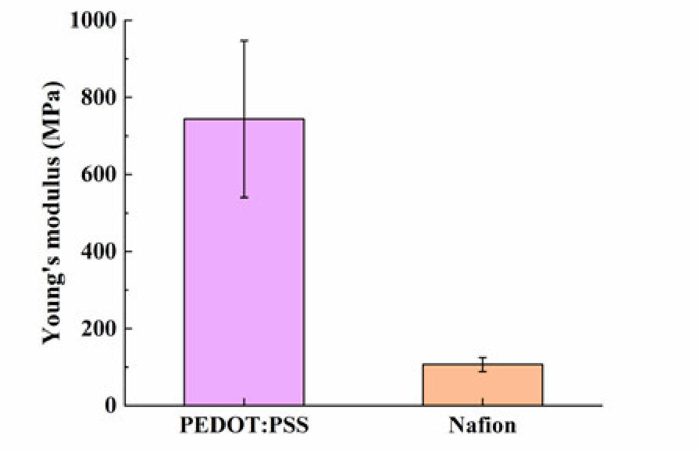


**Figure S2.** The Young's modulus of PEDOT:PSS and Nafion measured by AFM. The data are shown as mean ± SD.
